# Altruistic defence behaviours in aphids

**DOI:** 10.1186/1471-2148-10-19

**Published:** 2010-01-20

**Authors:** Gi-Mick Wu, Guy Boivin, Jacques Brodeur, Luc-Alain Giraldeau, Yannick Outreman

**Affiliations:** 1 Department of Natural Resource Sciences, McGill University, 21,111 Lakeshore Road, Ste-Anne-de-Bellevue, QC, H9X 3V9, Canada; 2 Centre de recherche et développement en horticulture, Agriculture & Agroalimentaire Canada, 430 boul. Gouin, St-Jean-sur-Richelieu, QC, J3B 3E6, Canada; 3 Institut de Recherche en Biologie Végétale, Université de Montréal, 4101 Sherbrooke Est, Montréal, QC, H1X 2B2, Canada; 4 Département des sciences biologiques, Université du Québec à Montréal, Case postale 8888, succursale Centre-ville, Montréal, QC, H3C 3P8, Canada; 5 UMR 1099 INRA-Agrocampus Ouest-Université Rennes I "Biologie des Organismes et des Populations appliquée à la Protection des Plantes" [BIO3P], 65 rue de Saint-Brieuc CS 84215, 35042 Rennes Cedex, France

## Abstract

**Background:**

Altruistic anti-predatory behaviours pose an evolutionary problem because they are costly to the actor and beneficial to the recipients. Altruistic behaviours can evolve through indirect fitness benefits when directed toward kin. The altruistic nature of anti-predatory behaviours is often difficult to establish because the actor can obtain direct fitness benefits, or the behaviour could result from selfish coercion by others, especially in eusocial animals. Non-eusocial parthenogenetically reproducing aphids form colonies of clone-mates, which are ideal to test the altruistic nature of anti-predatory defence behaviours. Many aphids release cornicle secretions when attacked by natural enemies such as parasitoids. These secretions contain an alarm pheromone that alerts neighbours (clone-mates) of danger, thereby providing indirect fitness benefits to the actor. However, contact with cornicle secretions also hampers an attacker and could provide direct fitness to the actor.

**Results:**

We tested the hypothesis that cornicle secretions are altruistic by assessing direct and indirect fitness consequences of smearing cornicle secretions onto an attacker, and by manipulating the number of clone-mates that could benefit from the behaviour. We observed parasitoids, *Aphidius rhopalosiphi*, foraging singly in patches of the cereal aphid *Sitobion avenae *of varied patch size (2, 6, and 12 aphids). Aphids that smeared parasitoids did not benefit from a reduced probability of parasitism, or increase the parasitoids' handling time. Smeared parasitoids, however, spent proportionately more time grooming and less time foraging, which resulted in a decreased host-encounter and oviposition rate within the host patch. In addition, individual smearing rate increased with the number of clone-mates in the colony.

**Conclusions:**

Cornicle secretions of aphids were altruistic against parasitoids, as they provided no direct fitness benefits to secretion-releasing individuals, only indirect fitness benefits through neighbouring clone-mates. Moreover, the use of cornicle secretions was consistent with their altruistic nature, because the occurrence of this behaviour increased with the size of indirect fitness benefits, the number of clone-mates that can benefit. This study provides evidence for a case of kin-directed altruistic defence outside eusocial animals.

## Background

The adaptive value of most anti-predatory behaviours is quite intuitive, as they lower the actor's risk of mortality due to predation. However, behaviours such as alarm signalling in birds and mammals [1], predator inspection by fish [2,3] or aggressive defences by worker honeybees [4], pose an evolutionary challenge, because the cost of these behaviours is born by the actor, while other individuals (recipients) benefit from them. In some cases, the actor also obtains selfish benefits that are enough to offset the costs of the behaviour, so that benefits to others may be incidental (mutual benefit or weak altruism) [5,6]. In more extreme cases of altruism, the actor incurs a net fitness cost and the behaviour can evolve through indirect fitness benefits if it is preferentially directed toward individuals (usually kin) who share the same genes [5,7,8]. In this paper, we refer to altruism as the latter, more extreme form of altruism.

In animals living in groups, many anti-predatory behaviours benefit individuals other than the actor, but few have been shown to be purely altruistic. Alarm signals, for instance, warn conspecifics of the presence of potential predators [1,9,10], but may be used selfishly to manipulate other group members [11] or to inform the predator that the actor is more difficult to catch than other individuals [12-14]. Likewise, predator inspection increases exposure to predation for the benefit of the group [3], but the actor may obtain better information and consequently escape attacks more easily than other individuals [15,16]. Aggressive defences are clearly costly to the actor and benefit recipients in the form of protection or an opportunity to escape [1]. Aggressive attacks, and other seemingly altruistic anti-predatory behaviours, can increase mating success of the actor [17], and survival of potential mates [18] or direct descendants [19] of the actor. Because mating success and the survival of direct offspring (parental care) provide the actor with direct fitness benefits, these behaviours can be qualified as mutually benefiting rather than altruistic [5]. In the case of eusocial animals, anti-predatory behaviours are performed by non-reproductive castes such as worker or soldier ants [20]. While these behaviours clearly benefit the reproductive queen(s) and not the actor, they may be the result of selfish control by the queen or by other individuals in the colonies [21,22].

Parthenogenetically reproducing animals that form groups of clone-mates such as aphids (Hemiptera: Aphididae) [23,24] provide the ideal system to test kin-selection of altruistic behaviours used against predators or insect parasitoids. Because these aphids are not eusocial, selfish manipulation by queens or workers is not a confounding factor. Additionally, other studies suggest that altruistic defences can evolve in aphids. For instance, pea aphids (*Acyrthosiphon pisum*) that have been parasitized tend to drop off their plant and increase their chances of dying, thereby decreasing parasitoid load for the following generations of aphids [25,26]. However, non-altruistic interpretations of this suicidal behaviour have been proposed [27,28].

Group living confers aphids with many anti-predatory benefits [1,29,30]. For instance, colonies of aphids create a dilution effect [31], which can also be enhanced with decoys by leaving empty exoskeletons after moulting [32] or by remaining near dead aphids [33]. In addition, most species of aphids also possess a pair of cornicles, which are projections that stick out of their abdomen [34]. When attacked by an enemy (i.e., a predator or an insect parasitoid), aphids can release sticky secretions [35] that contain an alarm pheromone [36] from the tips of these cornicles. The alarm pheromone of aphids, (E)-*β *-farnesene, elicits defensive or escape responses in neighbouring aphids [37-39] and increases their survival [40]. Because neighbours (recipients) are often clone-mates, the alarm function provides indirect fitness benefits to the aphid releasing the secretions (the actor). Furthermore, cornicle secretions are released in a super-cooled liquid form, which hardens upon contact with an object such as an enemy [35]. These hardening secretions hinder enemies and could reduce the risk of predation or parasitism for the aphid releasing them [41], thereby having a direct fitness benefit. A predator or parasitoid could also be temporarily or permanently incapacitated by the hardened secretions and unable to attack nearby aphids [42] or may decide to leave the colony prematurely [43]. This would provide the aphid releasing the secretions an indirect fitness benefit through increased survival of clone-mates.

Cornicle secretions are mostly composed of triglycerides [36] and therefore costly to produce for sap-feeding aphids, which lack lipids in their diet. The release of cornicle secretions reduces the amount of lipid available for development [44], reproduction or dispersal [45]. Releasing even a single droplet can delay or reduce reproduction of aphids, especially when doing so before attaining maturity [46]. Furthermore, releasing cornicle secretions can have an ecological cost as the volatiles contained in them may attract species of predators [47,48] and parasitoids [49,50].

Whereas the alarm function of cornicle secretions clearly provides indirect fitness benefits, the fitness consequences of their mechanical function (smearing) remains unclear. Determining whether the mechanical function of cornicle secretions provides a direct fitness benefit to an aphid is required to establish whether cornicle secretions can be considered altruistic as a whole.

In this study, we tested the hypothesis that the release of cornicle secretions by an aphid is altruistic, using the parasitoid *Aphidius rhopalosiphi *(Hymenoptera: Braconidae) foraging in colonies of grain aphids, *Sitobion avenae *(Hemiptera: Aphididae) in laboratory experiments. We tested for the presence of a direct fitness benefit of cornicle secretions by investigating the effects of smearing on the success of parasitoid attacks. We tested for the presence of indirect fitness benefits by determining whether a parasitoid's rate of oviposition within a colony varied with the frequency of smearing events in that colony. As the number of clone-mates that can benefit from an aphid's release of cornicle droplets increases, the indirect fitness benefit should also increase. Finally, we tested the prediction that cornicle secretions are released more readily when the indirect fitness benefit is greater by manipulating the number of clone-mates in the colony.

## Methods

### Study system

*Sitobion avenae*, a common aphid of cereal crops, is parasitized by the solitary parasitoid *A. rhopalosiphi*. When under attack, this aphid releases cornicle secretions, to which conspecifics respond by waving antennae, ceasing to feed, or escaping [38,51]. Specimens of the aphid and its parasitoid were collected from wheat (*Triticum aestivum*) fields in Rennes, France, and reared in the laboratory. The rearing of aphids was initiated from a single clonal individual, such that relatedness was at its highest. Colonies were kept at 20°C, with a relative humidity of 70 ± 10% and a 16L:8D photoperiod. Second instar aphids and one day old mated female wasps were used in the experiment. Prior to testing, female wasps were allowed to gain experience by ovipositing into three aphids on a single wheat leaf.

### Laboratory experiment

Female parasitoids (n = 50) were allowed to forage individually in a glass cage (40 × 30 × 50 cm) containing eight single-leafed wheat plants (15 cm tall). Three hours before behavioural observations, six of the eight plants were inoculated with 2, 6, or 12 clone-mates in equal proportions (two plants per aphid density). The two remaining plants were not infested. This range of patch density is commonly encountered by the parasitoid females under natural conditions. Empty patches (0 aphids) were not included in analyses, as no aphid-parasitoid interactions could be observed in them. Each parasitoid was introduced individually at the centre of the cage and observed continuously until it had either visited all eight patches (i.e., the eight wheat plants) or two hours had elapsed. For each patch visit, the observer recorded the number of aphids in the patch, as well as the behaviour of the parasitoid on that patch. Behaviours were defined as *searching *(walking on the plant), *stationary *(immobile), *grooming *(often to remove cornicle secretions), *oviposition *(encountering and stinging a host), and *rejection *(encountering a host without stinging). Stinging was assumed to result in oviposition, because *A. rhopalosiphi *cannot distinguish freshly parasitized from unparasitized hosts [52]. Smearing of the parasitoid with cornicle secretions during an attack was also recorded. The timing of events was recorded with a 0.1 s precision using "The Observer 3.0" (Noldus Information Technology, Wageningen, The Netherlands).

### Statistical Analyses

We assessed the presence of direct fitness benefits to the actor by determining whether smearing during an attack was associated with a lower probability of being stung. When aphids were stung, we also tested the effect of smearing during an attack on the parasitoid's handling time, because longer handling times may increase the probability that a parasitoid will give up or be interrupted. For each analysis, we included patch density (2, 6, 12 aphids/patch) and timing of attacks (time spent in the patch) as covariates.

We assessed the potential indirect fitness benefits of smearing by measuring the effects of smearing frequency on the parasitoid's oviposition rate (number of ovipositions offset by patch time) in the patch. We also included patch density as a covariate. To understand how smearing affects oviposition rate, we tested the effect of smearing frequency on components of the parasitoid's oviposition rate: encounter rate (number of encounters offset by patch time); the outcome of host encounters (oviposition vs. rejection); average handling time of ovipositions (time spent in oviposition offset by the number of ovipositions); and the proportion of time spent foraging (searching for and handling hosts), grooming, and stationary (duration of behaviours offset by patch time). Aphid density was included as a covariate. Finally, we evaluated the effect of aphid density on smearing rate (smearing frequency offset by patch time), and included the number of host encounters as a covariate. We included second order interaction terms in all statistical analyses, but present only significant interactions.

We used generalized estimating equations to account for the different families of distributions of the dependent variables and the multiple observations per subject [53]. The statistical analyses used binomial (logit link function), Poisson (log link function), and gamma (log link function) error terms for the outcome of encounters (oviposition vs. rejection), frequencies of behaviours, and handling times, respectively. We specified "independence" (no correlation) as the working correlation structure for within-subject observations, because the true correlation structure was unknown [54]. We tested the robustness of this specification by comparing the fit of each statistical model using two other common working correlation structures: "exchangeable" (fixed correlation within individual) and "auto-regressive (1)" (correlation increasing with proximity between observations) using the QIC information criterion [54]. The independence working correlation structure almost always produced the best fit. When a different correlation structure produced the best fit, results of the analyses did not differ using the different working correlation structure.

We included only data from patch visits in which the parasitoid oviposited in at least one aphid in order to reduce any effects of patch depletion. When analysing patch data, we excluded those with three or four occurrences of smearing, because such frequencies were extremely rare (5 and 1 patch visits, respectively) and occurred only in patches of 12 aphids. Analyses were conducted using the package "geepack" version 1.0-16 [55,56] in R version 2.9.0 [57].

## Results

We gathered over 66 hours of observations for the 50 foraging parasitoids. Observations consisted of 326 patch visits that included ovipositions. Patch visits lasted approximately 6.7 minutes (median). We observed a total of 6019 encounters of which 1837 (31%) resulted in oviposition, and 132 (2%) included smearing of the parasitoid with cornicle secretions.

### Direct fitness benefits of smearing

**Figure 1:**
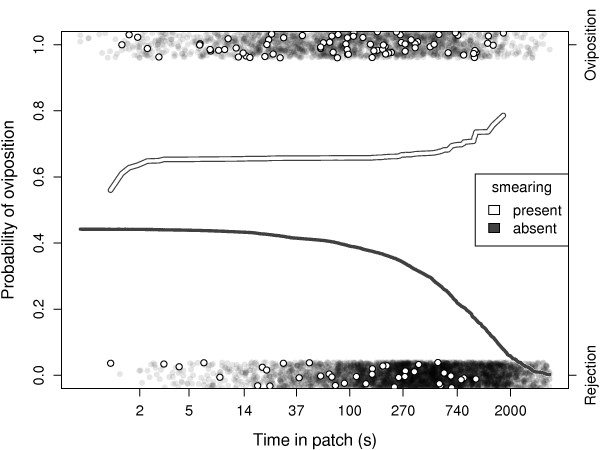
**Effect of smearing on the outcome of encounters between parasitoids and aphids**. Lines show the fitted probabilities of oviposition and dots show the outcome of individual encounters (oviposition vs. rejection), as a function of the timing of events. Encounters resulted in oviposition more frequently in the presence of smearing (white), than in its absence (grey).

Smearing did not reduce an aphid's probability of being the victim of an oviposition (Wald = 2.64, df = 1, p = 0.10). On the contrary, aphids that smeared their attacker had a higher probability of being a victim of oviposition during the later part of patch visits (interaction: smearing × timing of events, Wald = 8.59, df = 1, p = 0.0034; Figure 1). Aphid density did not affect the outcome of individual encounters (Wald = 1.33, df = 1, p = 0.25). Handling time was similar for ovipositions during which the parasitoid was smeared (median = 6.7 s) or not (median = 4.4 s) (Wald = 0.09, df = 1, p = 0.77). Aphid density did not affect handling time of ovipositions (Wald = 0.55, df = 1, p = 0.46).

### Indirect fitness benefits of smearing

**Figure 2:**
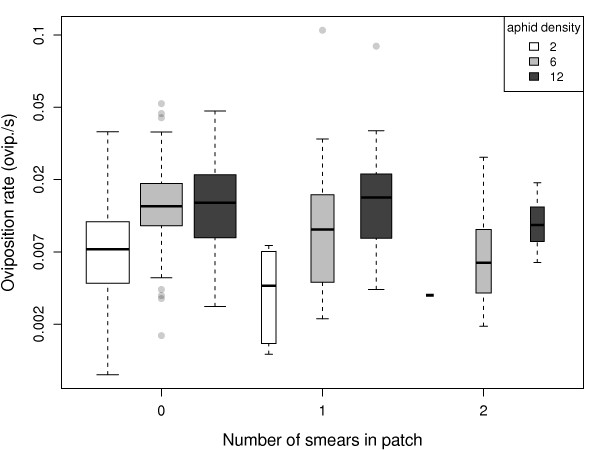
**Parasitoid oviposition rate within patch visits against smearing frequency**. Box plots show the distribution of oviposition rates for patches containing 2 (white), 6 (grey), and 12 (black) aphids. Boxes show the inter-quartile range (50% of observations) in which the horizontal bar is the median. Whiskers extend 1.5 times the interquartile range beyond the median. Dots show individual observations lying outside this interval. Box widths are proportional to the square root of sample sizes.

**Figure 3:**
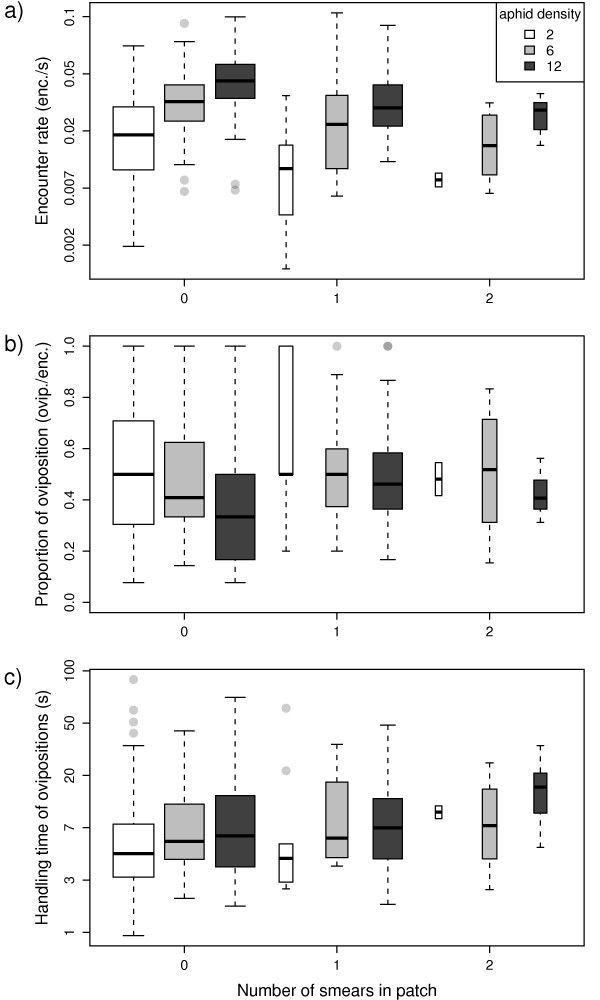
**Components of the parasitoids' foraging success within patch visits against smearing frequency**. Box plots show the distributions of: a) encounter rate; b) outcome of encounters; and c) handling time of ovipositions for patches containing 2 (white), 6 (grey), and 12 (black) aphids. Encounter rate (a) and handling time (c) are plotted on a log scale.

Oviposition rate within patches (Figure 2) decreased significantly with increasing smearing frequency (Wald = 13.1, df = 1, p = 0.0003), and increased significantly with aphid density (Wald = 42.3, df = 1, p < 0.0001). Encounter rate with aphids (Figure 3a) decreased significantly with increasing smearing frequency (Wald = 11.5, df = 1, p = 0.0007), but increased significantly with aphid density (Wald = 66.7, df = 1, p < 0.0001). The proportion of encounters resulting in oviposition (Figure 3b) did not vary significantly with smearing frequency (Wald = 0.03, df = 1, p = 0.87) or aphid density (Wald = 1.84, df = 1, p = 0.17). Similarly, handling time of ovipositions in a patch (Figure 3c) did not vary significantly with smearing frequency (Wald = 0.27, df = 1, p = 0.60) or aphid density (Wald = 0.16, df = 1, p = 0.69).

**Figure 4:**
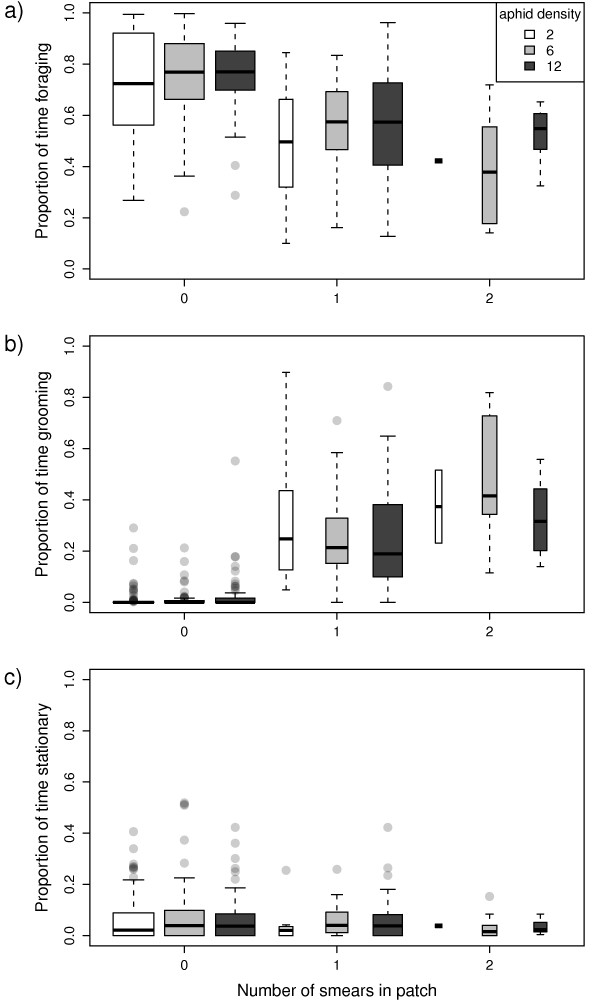
**Parasitoid's time budget within patch visits against smearing frequency**. Box plots show the proportion of time spent: a) foraging (searching & handling); b) grooming; and c) stationary in patches containing 2 (white), 6 (grey), and 12 (black) aphids.

The time budget of the parasitoids consisted mostly of foraging and grooming, while little time was generally spent stationary (Figure 4). The proportion of time spent foraging in the patch (Figure 4a) decreased significantly with smearing frequency (Wald = 5.0, df = 1, p = 0.025), but not with aphid density (Wald = 0.90, df = 1, p = 0.342). In contrast, the proportion of time spent grooming (Figure 4b) increased with smearing frequency (Wald = 50.5, df = 1, p < 0.0001), but was also unaffected by aphid density (Wald = 2.18, df = 1, p = 0.14). The proportion of time spent stationary (Figure 4c) was not affected by smearing frequency (Wald = 2.15, df = 1, p = 0.14) or aphid density (Wald = 0.01, df = 1, p = 0.93).

### Effect of the number of clone-mates on the occurrence of smearing

**Figure 5:**
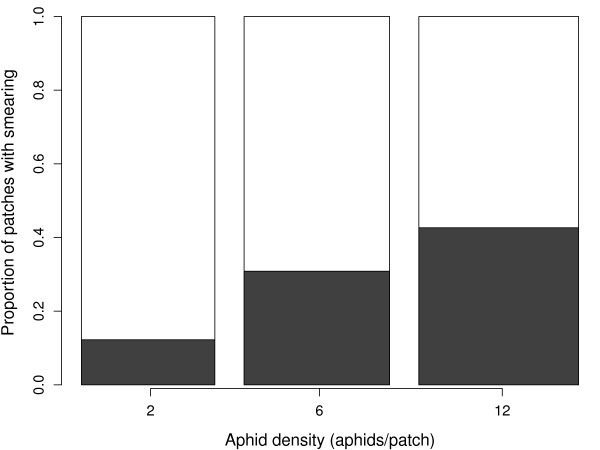
**Occurrence of smearing in patch visits against aphid density**. Dark area shows the proportion of patches in which the parasitoid was smeared. The widths of columns are proportional to the square root of sample sizes.

The proportion of patches in which smearing was observed (Figure 5) increased significantly with aphid density (Wald = 5.49, df = 1, p = 0.019). In one patch visit lasting only 7 s (aphid density = 6), the parasitoid oviposited in an aphid, was smeared by an aphid, and immediately left the patch. This resulted in a very high smearing rate, but excluding this patch visit did not affect the relationship between the proportion of patches with smearing and aphid density (Wald = 5.60, df = 1, p = 0.018). The proportion of patches with smearing did not increase with the number of encounters in the patch (Wald = 0.02, df = 1, p = 0.88).

## Discussion

The act of smearing parasitoids with cornicle secretions can be considered altruistic, because it does not reduce the actor's probability of being parasitized, but reduces the parasitoid's rate of oviposition in the colony kin. Moreover, the occurrence of smearing increased with the number of clone-mates in the colony, which is consistent with kin-directed altruism. We discuss these components of altruism in turn.

### Absence of direct fitness benefits

For *S. avenae*, there is no direct fitness benefit from smearing the parasitoid *A. rhopalosiphi*, as our detailed analysis found no reduction in the probability of parasitism associated with smearing. On the contrary, aphids that smeared parasitoids were more likely to be parasitized than others, when smearing occurred late in the exploitation of a patch. The absence of direct fitness benefits may be due to smearing occurring once it is too late to prevent parasitism. The specific oviposition behaviour of the parasitoid may contribute to the lack of any direct fitness benefit of smearing. Like many parasitoids of the subfamily Aphidiinae, *A. rhopalosiphi *can sting an aphid and deposit its egg in less than a second [58]. The duration of the entire attack sequence, from the encounter to the end of oviposition, was very short (median = 4.4 s) and left little time for the aphid to disrupt its attacker and prevent parasitism.

### Evidence of indirect fitness benefits

A parasitoid that has been smeared by a host incurs a reduced oviposition rate within a patch, because it spent less time foraging and more time grooming to remove the hardened cornicle secretions from its body. This, in addition to the effect of the alarm pheromone (which causes neighbouring aphids to express defence behaviours such as kicking, walking away and dropping from the leaf), reduces the parasitoid's foraging efficiency [59]. The combined alarm and smearing functions of cornicle secretions provided a considerable benefit for other aphids in the colony, because alerted aphids run away and become unavailable to the parasitoid while it was busy grooming. Consequently, even small increases in grooming time or small reductions in oviposition rate can be costly in terms of lost opportunity cost [60]. Further studies could aim at quantifying the specific contribution of alarm and smearing mechanism to the reduction of parasitism rate in aphid colonies, and determine whether these effects are additive or synergistic.

Smearing has also been shown to benefit an entire colony of *S. avenae *by reducing the patch residence time of *A. rhopalosiphi *[43]. Our study is complementary in showing a benefit to the aphid colony while the parasitoid remains within the patch. This decrease in foraging rate within the patch may be responsible for the shorter patch residence time reported by Outreman and co-workers [43] if the parasitoid was foraging optimally [61,62]. An explicit test of this prediction, however, should consider a possible change in the shape of the parasitoid's fitness gain curve [63] that could result from the long periods of grooming following smearing events.

Increasing the number of clone-mates in the colony increases the magnitude of indirect fitness benefits, because a greater number of clone-mates can benefit from smearing. As expected, smearing occurred more frequently when a greater number of clone-mates were present. This result was not simply due to a greater number of encounters or a longer patch residence time in colonies containing more individuals, because our analyses controlled for both. A similar increase in altruistic behaviour with increasing number of kin is found in alarm calls of some birds and mammals [1,64]. Further evidence for the altruistic nature of cornicle secretions could be obtained by varying the relatedness of the recipients as is usually done in other systems [65]. In colonies of parthenogenetically reproducing aphids, however, individuals are usually clone-mates, so that the number of individuals may be more important than relatedness. Aphids may therefore not have evolved the ability to discriminate kin from conspecifics, as suggested in a recent study of pheromone production in the absence of predators [47].

### Evolution of cornicle secretions

Our results are consistent with the hypothesis that the use of cornicle secretions by *S. avenae *against *A. rhopalosiphi *is altruistic and is maintained through kin selection. Cornicle secretions, however, may not have evolved specifically against aphidiine parasitoids, as aphids are preyed upon by larvae of syrphid flies, predatory midges, coccinellids and other invertebrates, and are hosts for other aphidiine and aphelinid wasps. For most aphidiine parasitoids, attacks and ovipositions may be quick enough to preclude any direct fitness benefits to the aphid releasing cornicle secretions. The slower aphelinid wasps [66] and predators which need to grasp and kill their victims, however, may be more exposed to smearing. For instance, pea aphids (*Acyrthosiphon pisum*) that defend against coccinellid predators using cornicle secretions obtain both a direct [41] and an indirect fitness benefit [40]. For pea aphids, cornicle secretions could be better described as mutually benefiting rather than altruistic. Similarly, alarm calls in some birds and mammals may be selfish, benefit a group, or be kin selected, depending on the social context and the type of predator [1,64]. In addition, cornicle secretions may be maintained by altruism, but have evolved for selfish benefits. In rodents for instance, the evolution of alarm calls was likely selfish despite evidence of altruism in some species [12]. Tracing back the evolutionary history of cornicle secretions in aphids and their associated natural enemies may help determine whether this behaviour initially evolved through selfish or altruistic benefits.

The evolution of altruism can be constrained if the additional offspring of the recipients compete strongly with those of the altruist [67]. In fig wasps for instance, brothers compete exclusively among themselves to mate sisters and fight each other to death [68]. Increased competition is not a likely constraint in the case of cornicle secretions, because the alarm pheromone also increases the proportion of dispersal morphs in the next generation [69], thereby reducing competition between offspring of the recipient and those of the altruist.

## Conclusions

Our study provides evidence for a case of kin-directed altruistic defence in the grain aphid by showing that cornicle secretions, which are known to be costly, provide no direct fitness benefits to the actor, but instead provide indirect fitness benefits through kin. Moreover, the use of cornicle secretions was consistent with an altruistic behaviour as it increased when the number of clone-mates benefiting from it, and hence the indirect benefit, was greater. This constitutes one of the few examples of kin-directed altruistic defences outside eusocial systems.

## Authors' contributions

GMW contributed to design the research, analyzed the data and prepared the manuscript. GB, JB and LAG contributed to the manuscript. YO designed and conducted laboratory experiment and contributed to the manuscript. All authors read and approved the final manuscript.
